# Life-threatening hypersensitivity pneumonitis induced by docetaxel (taxotere)

**DOI:** 10.1054/bjoc.2001.2071

**Published:** 2001-09-01

**Authors:** G-S Wang, K-Y Yang, R-P Perng

**Affiliations:** Chest Department, Taipei Veterans General Hospital; School of Medicine, National Yang-Ming University, Taipei, Taiwan, ROC

**Keywords:** docetaxel, non-small-cell lung cancer, hypersensitivity pneumonitis

## Abstract

4 patients with advanced non-small-cell lung cancer (NSCLC) treated with docetaxel developed life-threatening pneumonitis requiring mechanical ventilation. Docetaxel (30–60 mg m^−2^, according to a different protocol) was infused within one hour with standard premedications. One patient's pneumonitis occurred 5 days after the first dose of docetaxel, and that of the other 3 between the 2nd and 6th cycles. Based on the clinical course, radiological findings of an interstitial pneumonitis, and exclusion of other possible resultant causes, including metastatic cancer, radiation pulmonary injury, infection, or connective tissue disease, hypersensitivity pneumonitis was diagnosed. The patients were treated with hydrocortisone at 1200 mg per day or methylprednisolone at 240 mg per day. Although 3 of the 4 had a partial improvement in lung oxygenation, all patients’ conditions of hypersensitivity pneumonitis persisted and were complicated by other events, such as hospital-acquired infection and tension pneumothorax. The presence of this unusual hypersensitivity pneumonitis, which was so severe as to be life-threatening and refractory to high-dose corticosteroid therapy, should be taken into account during docetaxel treatment. © 2001 Cancer Research Campaign


*British Journal of Cancer* (2001) **85**(9), 1247–1250
© 2001 Cancer Research Campaign
doi: 10.1054/ bjoc.2001.2071, available online at http://www.idealibrary.com on IDEAL
http://www.bjcancer.com

### Life-threatening hypersensitivity pneumonitis induced by docetaxel (taxotere)

**G-S Wang, K-Y Yang and R-P Perng**

Chest Department, Taipei Veterans General Hospital; School of Medicine, National Yang-Ming University, Taipei, Taiwan, ROC

**Summary** 4 patients with advanced non-small-cell lung cancer (NSCLC) treated with docetaxel developed life-threatening pneumonitis requiring mechanical ventilation. Docetaxel (30–60 mg $m^{-2}$, according to a different protocol) was infused within one hour with standard premedications. One patient's pneumonitis occurred 5 days after the first dose of docetaxel, and that of the other 3 between the 2nd and 6th cycles. Based on the clinical course, radiological findings of an interstitial pneumonitis, and exclusion of other possible resultant causes, including metastatic cancer, radiation pulmonary injury, infection, or connective tissue disease, hypersensitivity pneumonitis was diagnosed. The patients were treated with hydrocortisone at 1200 mg per day or methylprednisolone at 240 mg per day. Although 3 of the 4 had a partial improvement in lung oxygenation, all patients' conditions of hypersensitivity pneumonitis persisted and were complicated by other events, such as hospital-acquired infection and tension pneumothorax. The presence of this unusual hypersensitivity pneumonitis, which was so severe as to be life-threatening and refractory to high-dose corticosteroid therapy, should be taken into account during docetaxel treatment. © 2001 Cancer Research Campaign

**Keywords**: docetaxel; non-small-cell lung cancer; hypersensitivity pneumonitis

The taxanes are a novel family of anti-cancer drugs, which has developed clinical significance in the past two decades; they are used mainly in advanced or metastatic breast and non-small-cell lung cancer (NSCLC) (Vaishampayan et al, 1999; Munster and Judis, 2000). Unlike other tubuline-binding agents such as the vinca alkaloids, which promote microtubule disassembly, the taxanes stabilize microtubules by preventing depolymerization, resulting in cell death. There are 2 structure-related members in the taxane family: paclitaxel and docetaxel.

Paclitaxel (taxol) was originally a natural product derived from the bark of the North American yew tree, *Taxus brevufola*. The chief adverse effects of paclitaxel are hypotension, arrthymia, neutropenia, peripheral neuropathy, nausea with vomiting, pneumonitis, and hypersensitivity reaction (Tamura et al, 1995; Furuse et al, 1997; Choy et al, 1998); the last may be attributed to both paclitaxel and its diluent, and can be reduced by premedications with steroid and histamine receptor antagonists.

Docetaxel (taxotere) is a chemically semi-synthesized compound derived from the extract of the needles of the European yew, *Taxus baccata*, and entered clinical trials in the 1990s. The chief adverse effects of docetaxel are neutropenia, hypersensitivity reaction, peripheral neuropathy, typhlitis, peripheral oedema, and very rarely, pneumonitis (Kunitah et al, 1996; Earhart, 1999; Fossella, 1999; Ibrahim et al, 2000).

Pneumonitis is an unusual side effect of the taxanes; there have been few reports about it. Phase II trials of paclitaxel disclosed an approximately 3% incidence rate of interstitial pneumonitis in patients with previously untreated advanced NSCLC (Furuse et al, 1997), and a 12% rate in patients with locally advanced NSCLC receiving concurrent radiotherapy (Choy et al, 1998). The precise mechanism of pneumonitis is not well-known, but may be related to hypersensitivity pneumonitis and precipitated by chest radiation (Schweitzer et al, 1995; Khan et al, 1997; Fujimori et al, 1998), since the taxanes, both paclitaxel and docetaxel, are potent radiosensitizers.

Only 6 cases, in which patients with advanced NSCLC treated with docetaxel suffered from acute interstitial pneumonitis, are mentioned in the literature (Kunitah et al, 1996; Merad et al, 1997; Etienne et al, 1998); of these, 5 patients recovered with corticosteroid treatment and one died of respiratory failure. The incidence rate was around 3%, as described in a small phase II series (Kunitah et al, 1996).

We report 4 cases of advanced NSCLC developing acute life-threatening bilateral interstitial pneumonitis and respiratory failure after 1–6 courses of docetaxel therapy, and discuss the phenomenon.

### CASE REPORTS

### Case 1

A 44-year-old female was diagnosed in March 1999 with stage IV adenocarcinoma of the lung, right lower lobe, with lung-to-lung metastases, lymphangitic spreading, and multiple bone metastases. She received local palliative radiotherapy with 3000 cGy to the lumbar 4–5 spine and left sacroiliac joint, followed by 6 courses of chemotherapy with cisplatin and etoposide, ending in August 1999. Failure of treatment led to her further receiving second-line chemotherapy with weekly docetaxel. The first dose of docetaxel, at 44 mg (33 mg $m^{-2}$), was infused within one hour on 20 September 1999, with premedications of steroid and anti-emesis agents. She developed severe shortness of breath and was sent to

*Received 7 December 2000*
*Revised 16 May 2001*
*Accepted 20 July 2001*

*Correspondence to:* Dr. K-Y Yang our emergency department 5 days later. The chest X-ray at that time revealed acute diffuse interstitial infiltrates, and marked hypoxemia was observed. She was then intubated with mechanical ventilatory support and immediately admitted to the intensive care unit; the post-intubation arterial blood analysis revealed that the arterial partial pressure of oxygen ($PaO_2$) was 166.9 mmHg, at a fraction of inspired oxygen ($FiO_2$) 0.5.

Empiric broad-spectrum intravenous antibiotics were used initially because of her moderate-grade fever (38.5˚C) and blood leukocytosis (WBC 130 00 $cumm^{-1}$), without a significant bacterial culture result. Her condition progressively became worse, and the $PaO_2$ was 72 mmHg at $FiO_2$ 0.5 by the 5th day of admission. Intravenous hydrocortisone at 300 mg every 6 hours was then prescribed for the suspicion of drug-induced pneumonitis. Blood oxygenation mildly improved ($PaO_2$ 73.2 mmHg at $FiO_2$ 0.35) in the following 10 days. However, when the dosage of corticosteroid was tapered off, blood oxygenation deteriorated. Nosocomial urosepsis and septic shock developed after 3 weeks of high-dose corticosteroid therapy, and her condition worsened thereafter. She died on October 29, 1999.

### Case 2

A 73-year-old male was diagnosed with stage IV NSCLC, right lower lobe, with bone metastases in May 1998. He received 6 courses of chemotherapy with vinorelbine (navelbine) and cisplatin, later followed by 3 courses of tamoxifen, ifosfamide, etoposide, and cisplatin (TIEP) because of progressive disease. In July 1999, TIEP was withdrawn because of neurologic toxicities, and he began to receive weekly docetaxel 53.7 mg (30 mg $m^{-2}$), and gemcitabine 1432 mg (800 mg $m^{-2}$) every 3 weeks. A total of 6 courses were finished on 5 November 1999. The cumulative dosage of docetaxel was 644.4 mg. Unfortunately, he developed shortness of breath with exacerbated weakness, and visited the emergency department 2 weeks after the last dose of docetaxel. The chest X-ray and chest computed tomography (CT) revealed acute diffuse thickening lobular septa with areas of ground glass opacities, which suggested drug-induced lung injury. He was intubated with mechanical ventilatory support for impending respiratory failure when the $PaO_2$ had reached 138.7 mmHg at $FiO_2$ 1.0.

He was admitted to the intensive care unit immediately and treated with intravenous hydrocortisone at 300 mg every 6 hours, but with little effect. A transbronchial lung biopsy (TBLB) performed on the 5th day of admission revealed chronic inflammation and interstitial fibrosis. The serum cytomegalovirus (CMV) antibody, legionella antibody/antigen, and anti-human immunodeficiency virus (HIV) antibody were all negative. Subsequently, he developed secondary pulmonary infection, sepsis, and multiple-organ failure syndrome. He died on the 9th day of admission.

### Case 3

A 70-year-old male was diagnosed with stage IIIa squamous cell lung cancer, left lower lobe, in May 1998. He refused surgical intervention and received 3 courses of paclitaxel and cisplatin, followed by 6 courses of gemcitabine and vinorelbine, ending in January 1999. Relapse of the disease was noted in March 1999. He accepted pulmonary radiotherapy with 5993 cGy, but the tumour continue to enlarge, and radiation pneumonitis occurred 4 months after finishing radiotherapy. Since the treatment had failed, he was

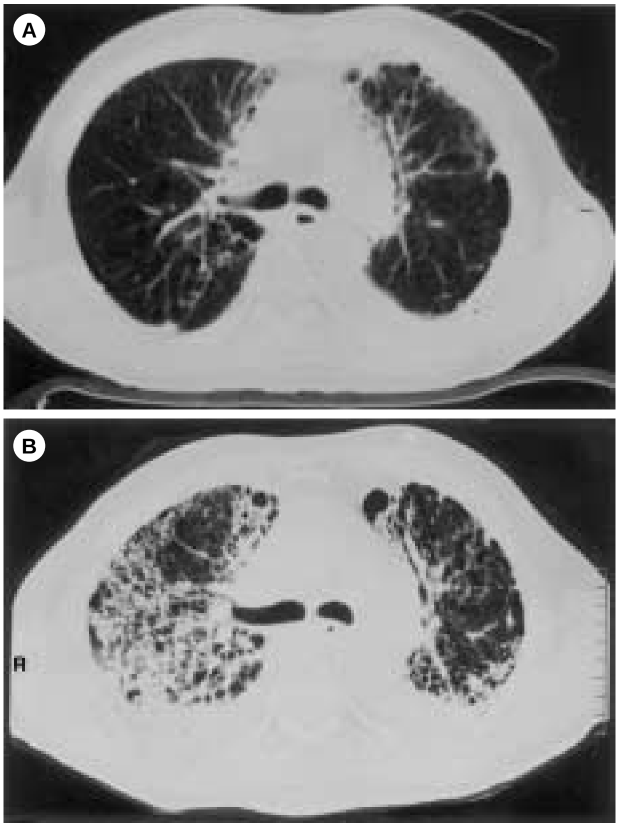

**Figure 1** Serial chest computed tomographs of case 3. (**A**) The chest computed tomograph was taken on November 5, 1999, when the 6 courses of chemotherapy with docetaxel and gemcitabine had just finished. This film shows mild fibrotic changes in both lungs that were compatible with radiation pneumonitis (6 months after finishing chest radiotherapy). (**B**). The chest computed tomograph was taken on November 30, 1999, one day after his last admission, when he developed severe hypoxemia and respiratory failure. This film shows coarse reticular interstitial and nodular infiltrations in the right lung field and left perihilar region that were compatible with hypersensitivity pneumonitis. Because of the rapid progression of the interstitial process, disseminated malignant disease or other systemic disease was excluded first; in addition to exposure history, clinical course, and laboratory data, including transbronchial lung biopsy, docetaxel-induced hypersensitivity pneumonitis was diagnosed then treated with weekly docetaxel 51 mg (30 mg $m^{-2}$), and gemcitabine 1360 mg (800 mg $m^{-2}$) every 3 weeks. A total of 6 courses were administered until 5 November 1999; the cumulative dosage of docetaxel was 612 mg. He developed dyspnoea on exertion and shortness of breath about 3 weeks after the last dose of chemotherapy, and rapidly deteriorated to impending respiratory failure on 29 November. He was intubated with mechanical ventilator support when the $PaO_2$ was 121.3 mmHg at $FiO_2$ 1.0, and was admitted to the intensive care unit.

The chest CT revealed increased interstitial infiltrates compared with the prior images (Figure 1). A TBLB performed 3 days after admission revealed chronic inflammatory changes. Docetaxel-induced pneumonitis was diagnosed. Intravenous methylprednisolone at 60 mg every 6 hours had been prescribed since admission. The blood oxygenation remained stationary in the initial few days; $PaO_2$ was 81 mmHg at $FiO_2$ 0.7. Unfortunately, nosocomial pneumonia with septic shock occurred one week later, and he died on the 12th day of admission.

*© 2001 Cancer Research Campaign*

### Case 4

A 75-year-old male was diagnosed with adenocarcinoma, right lower lobe, and underwent a right pneumonectomy in July 1999. Since the post-operation staging was stage IIIb ($N_3$), he received a subsequent 6 courses of chemotherapy with gemcitabine and cisplatin, ending in September 1999. Unfortunately, the disease progressed thereafter. He then received second-line chemotherapy with tri-weekly docetaxel 92.4 mg (60 mg $m^{-2}$) on 10 October and 74 mg (48 mg $m^{-2}$) on 31 October (the dosage was reduced to 80% due to neutropenic fever developing after the first dose). He developed dyspnoea 2 weeks after the second dose of docetaxel and rapidly progressed to severe hypoxaemia. He was intubated with mechanical ventilatory support and admitted to the intensive care unit on 30 November, when the $PaO_2$ was 77.6 mmHg at $FiO_2$ 0.9.

The chest X-ray revealed diffuse interstitial infiltrates. Intravenous hydrocortisone at 300 mg every 6 hours had been initiated since admission for the high suspicion of docetaxel-induced pneumonitis. Blood oxygenation improved during the initial 6 days, with a $PaO_2$ 66.7 mmHg at $FiO_2$ 0.4. Unfortunately, left tension pneumothorax occurred on the 10th admission day, and then a bronchopleural fistula developed. He died on the 16th admission day.

### DISCUSSION

Many cytotoxic drugs such as bleomycin, methotrexate, nilutamide and procarbazine, antibiotics such as penicillin, ampicillin and minocycline, cardiovascular drugs such as propanolol and amiodarone, and other drugs such as chlorpropamide, isoniazid and hydralazine, can induce hypersensitivity pneumonitis (Holoye et al, 1978; Cooper et al, 1986a,b; Akoun et al, 1989, 1990; Guillon et al, 1992). This usually occurs acutely, unrelated to the cumulative dose of the drug or the duration of therapy, and responds well to corticosteroid treatment. Occasionally, peripheral eosinophilia has been noted, involving about 10 to 20% of the white blood cells, and may signal a favourable prognosis with complete resolution (Cooper et al, 1986). The pathology has found patchy eosinophilic infiltrates affecting the distal air spaces, with a relative sparing of the proximal airways and an absence of immune deposits (Holoye et al, 1978). Bronchoalveolar lavage (BAL) in these patients revealed lymphocytic alevolitis, a reverse lymphocyte subpopulation ratio, and increased eosinophils (Akoun et al, 1989, 1990; Guillon et al, 1992; Khan et al, 1997; Merad et al, 1997; Fujimori et al, 1998), which suggested cell-mediated hypersensitivity immune reaction. These findings were similar to organic antigen-induced hypersensitivity pneumonitis (Keller et al, 1984; Helmers and Pisani, 1994: 155–182). The pathogenesis of the 2 reactions may be linked to some degree, but the latter is believed to be caused by a compartmentalized cell-mediated immune reaction with a relatively early onset after exposure.

Pneumonitis is a rare complication of the taxanes; in the past decade, there have been several case reports of paclitaxel-induced hypersensitivity pneumonitis (Schweitzer et al, 1995; Furuse et al, 1997; Khan et al, 1997; Choy et al, 1998; Fujimori et al, 1998). Pulmonary toxicity induced by docetaxel is more rare, with only 6 brief case reports reviewed in the literature (Kunitah et al, 1996; Merad et al, 1997; Etienne et al, 1998). The toxicity in these cases usually occurred after the 2nd to 4th course of chemotherapy, with or without premedications, and was relieved promptly with corticosteroid therapy, except for one patient who died of acute respiratory failure. One of these 6 patients had received a BAL study, which showed lymphocytic alveolitis (69%), a mild lymphocyte subpopulation ratio reverse (CD4/CD8 = 1), and increased neutrophils (18%) and eosinophils (12%) (Merad et al, 1997). One interesting point mentioned by Merad et al was that there is perhaps a positive relationship between the tumour response and pulmonary injury (Merad et al, 1997); in the 2 cases with docetaxel-induced pulmonary injury that they reported, both had at least a partial tumour response and one was alive, and free of disease, for 44 months after chest radiotherapy. This may suggest that docetaxel can induce the proliferation of cytotoxic T-cells against a specific pulmonary antigen expressed by the tumour (Merad et al, 1997).

A summary of our 4 patients' characteristics can be found in Table 1. The pulmonary injury presented with diffuse interstitial infiltrates, rapid progression, and poor responsiveness to broad-spectrum antibiotics therapy; metastatic tumour or infectious aetiology was not likely according to these presentations. After excluding other resultant factors, including other possible drug-induced hypersensitivity, connective lung disease, or an unusual infection such as CMV, HIV or legionella, docetaxel-induced hypersensitivity pneumonitis was diagnosed.

Case 3 had received curative radiotherapy of 5993 cGY over the anterior chest and mediastinum 6 months before the onset of respiratory failure. Radiation recall pneumonitis was not likely because of the long interval between radiotherapy and onset of respiratory failure (Schweitzer et al, 1995; Choy et al, 1998); more likely, the docetaxel-induced pulmonary injury was aggravated by previous radiation pneumonitis. The serial chest CT scans of case 3 are shown in Figure 1.

**Table 1** Summary of patients' characteristics

| | Case 1 | Case 2 | Case 3 | Case 4 |
|---|---|---|---|---|
| Age (years old) | 44 | 73 | 70 | 70 |
| Sex | F | M | M | M |
| Underlying lung disease | Stage IV adenocarcinoma | Stage IV NSCLC | Stage IIIA squamous cell lung cancer; radiation pneumonitis | Stage IIIB adenocarcinoma |
| Prior therapy | PVP × 6 cycles | NC × 6, TIEP × 3 cycles | GN × 6 cycles; chest radiotherapy | Pneumonectomy; GC × 6 cycles |
| Cumulative dosage of docetaxel | 44 mg | 644.4 mg | 612 mg | 166.4 mg |
| TBLB | NA | Chronic inflammation and interstitial fibrosis | Chronic inflammation | NA |

PVP: etoposide (VP-16), cisplatin; NC: vinorelbine (navelbine), cisplatin; GN: gemcitabine, vinorelbine (navelbine); GC: gemcitabine, cisplatin.
NA: not available. TBLB: transbronchial lung biopsy.

*© 2001 Cancer Research Campaign*

Lung oxygenation improved temporarily with corticosteroid therapy in cases 1 and 4, but it deteriorated again when we tried tapering off the corticosteroid. That the more severe pulmonary injury required prolonged corticosteroid therapy in our patients might be due to the high cumulative dosage of docetaxel (more than 600 mg in cases 2 and 3) and the more complicated underlying lung conditions (previous radiation pneumonitis in case 3, earlier pneumonectomy in case 4). In addition, all 4 patients had previously been treated with other chemotherapy agents (see Table 1), in comparison with the previously untreated patients reported in the literature.

The mechanism of drug-induced hypersensitivity pneumonitis is not well understood. Most researchers agree that it is the consequence of a cell-mediated immunologic reaction with pulmonary sequestration of activated specific lymphocytes. Drug-induced hypersensitivity pneumonitis is best diagnosed by a provocation test coupled with BAL (Akoun et al, 1989, 1990; Guillon et al, 1992). However, these were not performed in our 4 patients because of the life-threatening pneumonitis and respiratory failure.

The treatment of choice for drug-induced hypersensitivity pneumonitis is the withdrawal of the offending drug and administration of corticosteroid therapy, usually 30 to 60 mg of prednisolone per day for 2 to 3 weeks (Merad et al, 1997), or higher (60 to 240 mg per day) in more severe conditions such as acute respiratory failure (Jantz and Sahn, 1999), with a slow and careful tapering-off period.

In conclusion, we report 4 cases of advanced lung cancer developing life-threatening pulmonary injury, and requiring mechanical ventilation, after the 1st to 6th course of docetaxel infusion. Although there was no evidence from the BAL, or the provocation or lymphocyte migration tests, docetaxel-induced hypersensitivity pneumonitis was diagnosed by excluding the other possible aetiologies and considering the clinical course. Physicians should be alert to this unusual, and perhaps life-threatening, adverse effect of docetaxel, in order to begin counteracting treatment as soon as possible.

### REFERENCES

Akoun GM, Milleron BJ, Mayaud CM and Tholoniat D (1989) Provocation test coupled with bronchoalveolar lavage in diagnosis of propanolol-induced hypersensitivity pneumonitis. *Am Rev Respir Dis* **139**: 247–249

Akoun GM, Liote HA, Liote F, Rahman SG and Juntz D (1990) Provocation test coupled with bronchoalveolar lavage in diagnosis of drug (Nilutamide)-induced hypersensitivity pneumonitis. *Chest* **97**: 495–498

Choy H, Safran J, Akerley W, Graziano SL, Bogart JA and Cole BF (1998) Phase II trial of weekly paclitaxel and concurrent radiation therapy for locally advanced non-small-cell lung cancer. *Clin Cancer Res* **4**: 1931–1936

Cooper JR JAD, White DA and Matthay RA (1986a) Drug-induced pulmonary disease. Part 1: Cytotoxic drugs. *Am Rev Respir Dis* **133**: 321–340

Cooper JR JAD, White DA and Matthay RA (1986b) Drug-induced pulmonary disease. Part 2: Noncytotoxic drugs. *Am Rev Respir Dis* **133**: 488–505

Earhart RH (1999) Docetaxel (Taxotere): Preclinical and general clinical information. *Semin Oncol* **26**(Suppl 17): 8–13

Etienne B, Perol M, Nesme P, Vuillermoz S, Robinet G and Guerin JC (1998) Pneumopathie interstitielle diffuse aigue sous docetaxel (Taxotere). A propos de 2 observations. *Rev Mal Respir* **15**: 199–203. [French; abstract in English].

Fossella FV (1999) Docetaxel in the treatment of non-small-cell lung cancer: Review of single-agent trials. *Semin Oncol* **26**(Suppl 16): 17–23

Fujimori K, Yokoyama A, Kurita Y, Uni K and Saijo N (1998) Paclitaxel-induced cell-mediated hypersensitivity pneumonitis. Diagnosis using leukocyte migration test, bronchoalveolar lavage and transbronchial lung biopsy. *Oncology* **55**: 340–344

Furuse K, Naka N, Takada M, Kinuwaki E, Kudo S, Takada Y, Yamakido M, Yamamoto H and Fukuoka M (1997) Phase II study of 3-hour infusion of paclitaxel in patients with previously untreated stage III and IV non-small-cell lung cancer. *Oncology* **54**: 298–303

Guillon JM, Joly P, Austran B, Denis M, Akoun G, Debre P and Mayaud C (1992) Minocycline-induced cell-mediated hypersensitivity pneumonitis. *Ann Int Med* **117**: 476–481

Helmers RA and Pisani RJ (1994) Bronchoscopy. In Chaper 13: *Bronchoalveolar lavage*, Prakash UBS (ed). pp 155–182. Mayo Foundation, Published by Raven Press, Ltd., New York

Holoye PY, Luna MA, Mackay B and Bedrossian CWM (1978) Bleomycin hypersensitivity pneumonitis. *Ann Int Med* **88**: 47–49

Ibrahim JK, Sahin AA, Dubrow RA, Lynch RM, Michaud LB, Valero V, Buzdar AU and Hortobagyi GN (2000) Colitis associated with docetaxel-based chemotherapy in patients with metastatic breast cancer. *Lancet* **355**: 281–283

Jantz MA and Sahn SA (1999) Corticosteroids in acute respiratory failure. *Am J Respir Crit Care Med* **160**: 1079–1100

Keller RH, Swartz S, Schlueter DP, Bar-Sela S and Fink JN (1984) Immunoregulation in hypersensitivity pneumonitis: phenotypic and functional studies of bronchoalveolar lavage lymphocytes. *Am Rev Respir Dis* **130**: 766–771

Khan A, McNally D, Tutschka PJ and Bilgrami S (1997) Paclitaxel-induced acute bilateral pneumonitis. *Ann Pharmacother* **31**: 1471–1474

Kunitah H, Watanabe K, Onoshi T, Furuse K, Niitani H and Taguchi T (1996) Phase II trial of docetaxel in previously untreated advanced non-small-cell lung cancer: A Japanese cooperative study. *J Clin Oncol* **14**: 1649–1655

Merad M, Cesne AL, Baldeyrou P, Mesurolle B and Chevalier TL (1997) Docetaxel and interstitial pulmonary injury. *Ann Oncol* **8**: 191–194

Munster PN and Hudis CA (2000) Role of taxanes in adjuvant therapy. *Cancer inve* **18**: 32–38

Schweitzer VG, Juilard GJF, Bajada CL and Parker RG (1995) Radiation recall dermatitis and pneumonitis in a patient treated with paclitaxel. *Cancer* **76**: 1069–1072

Tamura T, Sasaki Y, Nishiwaki Y and Saijo N (1995) Phase I study of paclitaxel by three-hour infusion: hypotension just after infusion is one of the major dose-limiting toxicities. *Jpn J Cancer Res* **86**: 1203–1209

Vaishampayan U, Parchment RE, Jasti BR and Jussain M (1999) Taxanes: An overview of the pharmacokinetics and pharmacodynamics. *Urology* **54** (Suppl 6A): 22–29

 *© 2001 Cancer Research Campaign*
